# Increases in Anti-infective Drug Prices, Subsequent Prescribing, and Outpatient Costs

**DOI:** 10.1001/jamanetworkopen.2021.13963

**Published:** 2021-06-18

**Authors:** Junsoo Lee, Heesoo Joo, Brian A. Maskery, Jonathan D. Alpern, Chanhyun Park, Michelle Weinberg, William M. Stauffer

**Affiliations:** 1 Division of Global Migration and Quarantine, US Centers for Disease Control and Prevention, Atlanta, Georgia; 2 Department of Economics, University at Albany, State University of New York, Albany; 3 Center for Global Health and Social Responsibility, Department of Medicine, University of Minnesota, Minneapolis; 4 Department of Pharmacy and Health Systems Science, Northeastern University, Boston, Massachusetts; 5 Now with Health Outcomes Division, College of Pharmacy, The University of Texas at Austin, Austin

## Abstract

This cross-sectional study examines the association of prices for drugs to treat hookworm and pinworm with prescribing and prescription-filling behaviors and total outpatient treatment costs.

## Introduction

The mean wholesale prices of the antiparasitic drugs albendazole (400 mg; Albenza) and mebendazole (600 mg; Emverm) increased between 2010 and 2019 from $3.16 to $582 for albendazole and from $32 to $2853 for mebendazole.^1,2^ In this cross-sectional study, the association of antiparasitic drug prices with prescribing and prescription-filling behaviors and total outpatient treatment costs (TOCs) are investigated for hookworm and pinworm. These results are compared with those for *Clostridioides difficile*, which was selected because of similar outpatient characteristics and available number and specificity of drugs available.

## Methods

The protocol for this study was reviewed by the US Centers for Disease Control and Prevention and was granted exempt status. The study used data from a deidentified database. All results are presented in aggregate form, and specific patients were not identified; thus, informed consent was not required in accordance with 45 CFR §46. This study follows the Strengthening the Reporting of Observational Studies in Epidemiology (STROBE) reporting guideline.

Data were abstracted from IBM MarketScan Research Databases for patients with private insurance from 2010 to 2018. Standard-of-care (SOC) drugs for hookworm and pinworm include albendazole, mebendazole, and over-the-counter pyrantel pamoate. Ivermectin was included because it is frequently prescribed for hookworm, although it is inferior and non-SOC.^3^ For *C difficile*, oral vancomycin hydrochloride, fidaxomicin, and metronidazole are SOC drugs.^4^ Individuals with outpatient diagnosis codes for each infection were selected, and prescription drug and TOCs were analyzed descriptively over time using methods previously described (eAppendix in the Supplement).^1^

The 95% CIs were calculated and reported with the mean values of listed variables. To further evaluate the increase or decrease of proportions of those who were treated or treatment costs over time, Welch 1-tailed *t* test was used. *P* < .05 was chosen as the significance threshold. We used Stata/MP statistical software version 14.2 (StataCorp) for statistical analysis. Data analysis was performed from September 2019 to January 2020.

## Results

**Figure 1.**
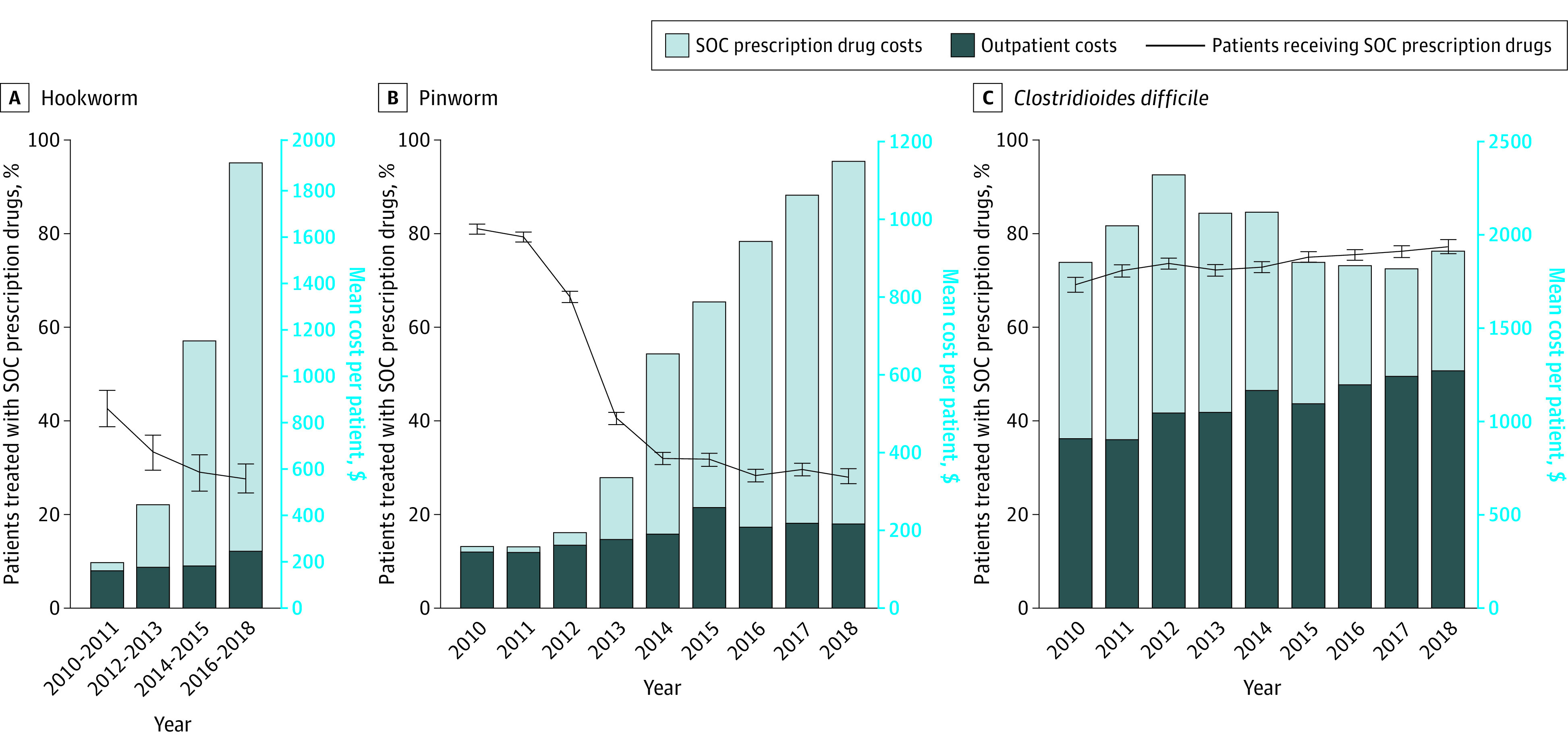
Proportions of Patients Treated With Standard-of-Care (SOC) Prescription Drugs and Mean Outpatient Visit and Drug Costs Numbers of patients with hookworm, pinworm, and *Clostridioides difficile *who were treated (and not treated) with SOC prescription drugs are as follows. For hookworm, the numbers are 267 (360) patients in 2010 to 2011, 206 (413) patients in 2012 to 2013, 156 (385) patients in 2014 to 2015, and 219 (572) patients in 2016 to 2018. For pinworm, the numbers are 4283 (1007) patients in 2010, 4915 (1286) patients in 2011, 4207 (2121) patients in 2012, 1917 (2810) patients in 2013, 1551 (3300) patients in 2014, 1322 (2849) patients in 2015, 1218 (3076) patients in 2016, 1234 (2940) patients in 2017, and 807 (2064) patients in 2018. For *C difficile*, the numbers are 2383 (1070) patients in 2010, 3393 (1320) patients in 2011, 4057 (1461) patients in 2012, 3663 (1418) patients in 2013, 4010 (1501) patients in 2014, 4188 (1403) patients in 2015, 4317 (1411) patients in 2016, 3625 (1136) patients in 2017, and 2502 (740) patients in 2018. The changes over time were tested by using the comparison between 2010 and 2018 data. Among the samples, the proportions of female patients were 52.2% for hookworm, 62.5% for pinworm, and 64.5% for *C difficile*. Mean ages for patients with hookworm, pinworm, and *C difficile* were 32.7, 13.1, and 42.0 years, respectively. Patients older than 65 years were excluded from the analysis to avoid issues with Medicare coinsurance payments, as mentioned in the eAppendix in the Supplement. Error bars denote 95% CIs.

**Figure 2.**
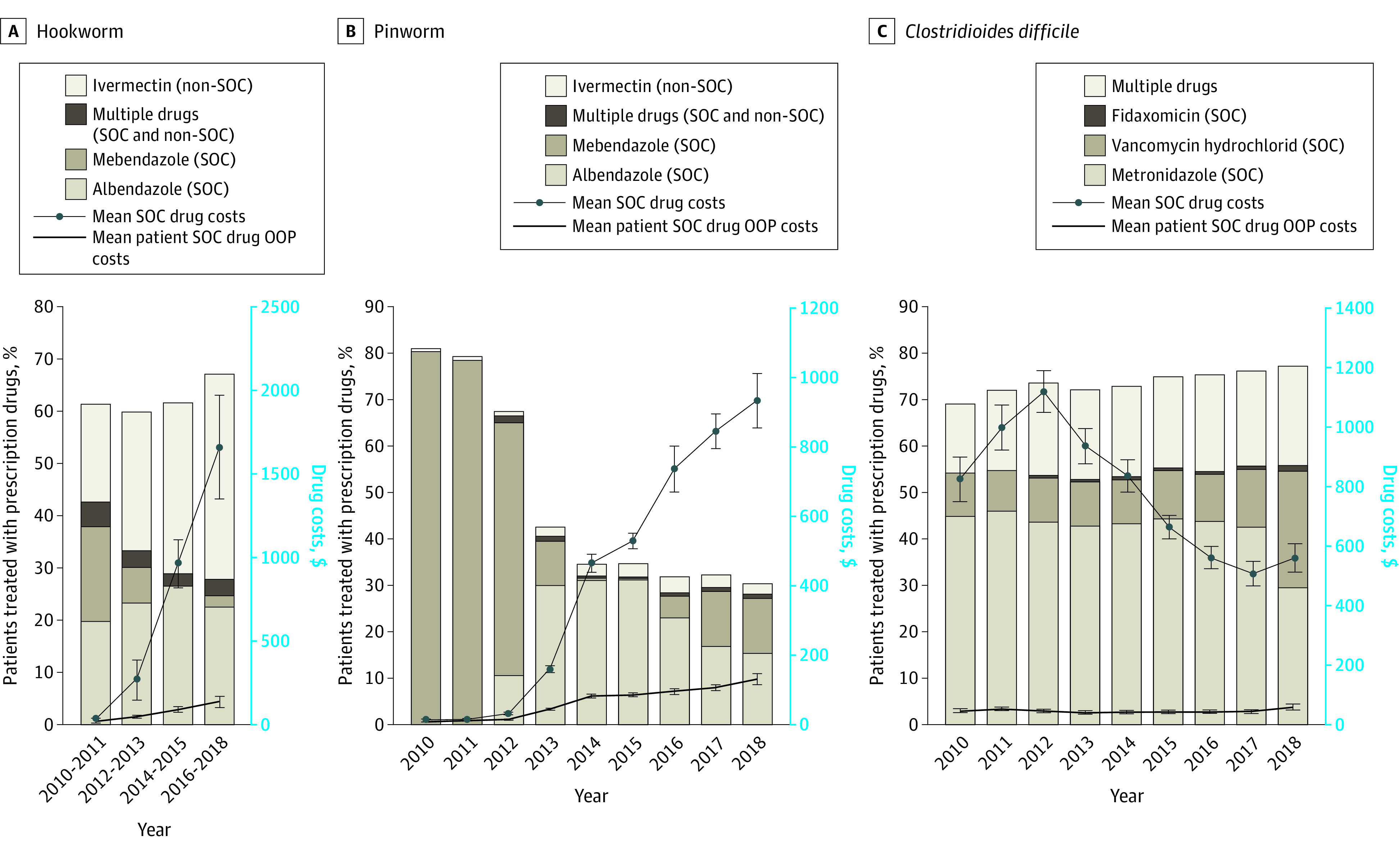
Proportions of Patients Treated With Standard-of-Care (SOC) Drugs or Non-SOC Prescription Drugs and Mean SOC and Patient Out-of-Pocket (OOP) Drug Costs The changes over time were estimated by using the comparison between 2010 and 2018 data. For hookworm and pinworm, the fractions filling prescriptions for ivermectin are shown. However, ivermectin is inferior to albendazole and mebendazole and is not considered an SOC treatment.^3^Error bars denote 95% CIs.

From 2010 to 2018, among 45 485 patients with a diagnosis of hookworm or pinworm, use of SOC drugs decreased as drug prices increased, whereas among 43 598 patients with *C difficile*, the use of SOC drugs increased as drug prices decreased (Figure 1). The use of SOC prescription drugs decreased from 43% to 28% of patients for hookworm and from 81% to 28% of patients for pinworm, while increasing from 69% to 77% of patients for *C difficile*. The mean (SD) SOC prescription drug costs for hookworm increased from $32.77 ($34.23) (median [interquartile range {IQR}], $27.12 [$13.37-$39.30]) to $1660 ($2333) (median [IQR], $1090 [$554-$1835]), and those for pinworm increased from $14.81 ($17.16) (median [IQR], $10.62 [$9.05-$16.01]) to $930 ($1134) (median [IQR], $743 [$480-$813]), with a corresponding out-of-pocket drug cost increase from $16.97 ($16.21) (median [IQR], $11.77 [$6.61-$25]) to $136 ($243) (median [IQR], $60 [$33.97-$121]) for hookworm and from $9.33 ($8.50) (median [IQR], $9.55 [$5.00-$10.74]) to $130 ($227) (median [IQR], $60 [$38-$125]) for pinworm (Figure 2). The medication cost contribution to TOCs increased from 15.2% to 88.6% for hookworm and from 9.4% to 81.2% for pinworm. For *C difficile*, the mean (SD) TOC was stable ($1844 [$4716] to $1904 [$3875]) (median [IQR], $444 [$123-$2271] to $636 [$231-$2194]), the mean (SD) out-of-pocket drug costs increased from $53 ($160) (median [IQR], $10 [$5-$40]) to $68 ($278) (median [IQR], $10.22 [$5-$30]), and the percentage contribution of medication to TOC decreased from 50.9% to 33.5% (Figure 1 and Figure 2). For parasitic infections, the proportion of patients who were prescribed ivermectin only (non-SOC) increased over time, especially for hookworm (Figure 2).

## Discussion

The extreme drug price increases for antiparasitics were associated with an increase in overall TOCs. A corresponding shift in prescribing from SOC to a non-SOC drug (ivermectin) was observed, suggesting decreased quality of care. By 2018, only 28% of patients with hookworm or pinworm received SOC prescriptions. No consistent non-SOC drug use for *C difficile* was observed. TOCs for hookworm and pinworm increased substantially, almost entirely accounted for by drug price increases. For *C difficile*, SOC treatment increased (from 69% to 77% of patients), whereas TOCs remained stable. Of note, the low use of mebendazole was associated with its withdrawal from the market in 2014 and 2015. It was reintroduced at a high price in 2016, after which use remained low. In addition, for *C difficile*, vancomycin became increasingly used as it emerged as the drug of choice beginning in 2017.^5^ These findings suggest that when there are limited treatment options, extreme drug price increases may be associated with higher patient treatment costs and lower use of recommended treatments. These results support previous findings.^6^

Limitations included that this was a descriptive study, there were inherent issues in reliance on claims data (eg, patients who purchased drugs without insurance claims), there was an absence of data for over-the-counter drugs, and there were potential unaccounted associated factors, such as formulary changes by insurance companies. In addition, the findings were limited to patients with private insurance.

To our knowledge, few published studies have directly assessed associations between drug prices and use.^6^ The findings of this cross-sectional study suggest that substantial drug price increases may be associated with higher patient treatment costs and lower use of recommended treatments.

## Notes

Supplement. **eAppendix. **Detailed Statistical Methods and *ICD* Codes eReferences
