# Supplementary material for: Increases in Anti-infective Drug Prices, Subsequent Prescribing, and Outpatient Costs
Source: JAMA Netw Open. 2021 Jun 18;4(6):e2113963. doi: 10.1001/jamanetworkopen.2021.13963 (PMC8214158; doi:10.1001/jamanetworkopen.2021.13963)
Supplement: Supplement. — eAppendix. Detailed Statistical Methods and ICD Codes eReferences [file jamanetwopen-e2113963-s001.pdf]

Supplemental Online Content

Lee J, Joo H, Maskery BA, et al. Increases in anti-infective drug prices, subsequent prescribing, and outpatient costs. *JAMA Netw Open*. 2021;4(6):e2113963. doi:10.1001/jamanetworkopen.2021.13963

eAppendix. Detailed Statistical Methods and *ICD* Codes

eReferences.

This supplemental material has been provided by the authors to give readers additional information about their work.

© 2021 Lee J et al. *JAMA Network Open.*

eAppendix. Detailed Statistical Methods and *ICD* Codes

Patients with a first diagnosis of hookworm, pinworm, or *Clostridioides difficile* (*C. diff*) from January 1, 2010 to December 31, 2018 in the IBM® MarketScan® Commercial Claims and Encounters (CCAE) database were selected using the Treatment Pathways web application. ICD-9-CM codes used to identify patients with hookworm infection were 126, 126.0, 126.1, 126.3, 126.8, 126.9 and ICD-10-CM codes used for hookworm infection were B76.0, B76.1, B76.8, B76.9. ICD-9-CM and ICD-10-CM codes for patients with pinworm infection were 127.4 and B80, respectively. For patients with *C. diff* infection, ICD-9-CM code 008.45 and ICD-10 codes A047 and A0472 were used.

We restricted the sample to include patients enrolled in private insurance only, excluding Medicare-eligible patients since Medicare payments were not included in the datasets. Patients needed to be continuously enrolled between 30 days before and 90 days after the first diagnosis. We included patients with non-capitated insurance and outpatient visits only (patients requiring hospitalization were excluded because these patients would have higher costs for different drug treatment regimens). For samples including patients with hookworm and pinworm infections, patients with co-infections with other parasitic diseases, such as ascariasis, schistosomiasis, strongyloidiasis, and trichuriasis, were excluded.

To identify standard of care (SOC) prescription drugs used for hookworm and pinworm, we followed the treatment guidelines from the U.S. Centers for Disease Control and Prevention (CDC).[1] CDC recommends using prescription-only albendazole and mebendazole for hookworm and pinworm treatment. CDC also recommends pyrantel pamoate,[2] which is an over-the-counter drug, for the treatment of pinworm and hookworm. Ivermectin, which is commonly used for the treatment of other parasitic diseases but is not recommended using the treatment of pinworm or

© 2021 Lee J et al. *JAMA Network Open.*

hookworm by CDC, was chosen to be analyzed as a non-SOC treatment of those two parasitic diseases because we observed that this drug was commonly used for treatment of patients with hookworm infections. For *C. diff*, vancomycin hydrochloride, fidaxomicin, and metronidazole were selected as SOC prescription drugs.

Among selected patients, outpatient visit costs, SOC prescription drug costs, and SOC prescription drug out-of-pocket (OOP) costs were calculated for patients who had SOC prescription drug claims. For each patient, our analysis included outpatient claims with the selected diagnosis codes for up to 90 days after the first diagnosis and SOC prescription drug claims that occurred between 30 days before and 90 days after the first diagnosis.

Using a descriptive study design, we estimated mean values of outpatient costs, excluding any outpatient drug costs, SOC outpatient prescription drug costs, and SOC OOP costs for hookworm, pinworm, and *C. diff*. Results were reported by time period. The proportions of patients who were treated with any SOC drugs, the proportions of patients who were treated with each SOC drug, and the proportions of those who were treated with multiple drugs were also reported. For patients with hookworm infection or pinworm infection, the proportions of patients who were treated with the non-SOC drug, ivermectin, reported. When assessing the proportions of hookworm and pinworm patients who were treated with multiple drugs, we included those patients who were treated with SOC drugs only in addition to those treated with an SOC drug and a non-SOC drug. No consistent non-SOC drug use for *C. diff* was identified in our sample.

The 95% confidence intervals were calculated and reported with the average values of listed variables. To further evaluate the increase or decrease of proportions of those who were treated or treatment costs over time, Welch's one-tailed t-test was used. A p-value<0.05 was chosen as the significance threshold.

© 2021 Lee J et al. *JAMA Network Open.*

eReferences

[1]Division of Parasitic Diseases, U.S. CDC. Parasites - Hookworm. Resources for Health Professionals. 2019 [cited 2021 April 15]; Available from: https://www.cdc.gov/parasites/hookworm/health_professionals/index.html#tx.
[2]Division of Parasitic Diseases, U.S. CDC. Parasites - Pinworm. Treatment. 2016 [cited 2021 April 15]; Available from: https://www.cdc.gov/parasites/pinworm/treatment.html.

© 2021 Lee J et al. *JAMA Network Open.*
